# Soil organic matter, rather than temperature, determines the structure and functioning of subarctic decomposer communities

**DOI:** 10.1111/gcb.16158

**Published:** 2022-03-21

**Authors:** Sinikka I. Robinson, Eoin J. O’Gorman, Beat Frey, Marleena Hagner, Juha Mikola

**Affiliations:** ^1^ Ecosystems and Environment Research Programme University of Helsinki Helsinki Finland; ^2^ 2591 School of Life Sciences University of Essex Colchester UK; ^3^ Swiss Federal Research Institute WSL Birmensdorf Switzerland; ^4^ Natural Resources Institute Finland (Luke) Jokioinen Finland; ^5^ Natural Resources Institute Finland (Luke) Helsinki Finland

**Keywords:** climate change, decomposition, ecosystem functioning, N mineralization, natural experiment, plant biomass, soil organic matter, structural equation model

## Abstract

The impacts of climate change on ecosystem structure and functioning are likely to be strongest at high latitudes due to the adaptation of biota to relatively low temperatures and nutrient levels. Soil warming is widely predicted to alter microbial, invertebrate, and plant communities, with cascading effects on ecosystem functioning, but this has largely been demonstrated over short‐term (<10 year) warming studies. Using a natural soil temperature gradient spanning 10–35°C, we examine responses of soil organisms, decomposition, nitrogen cycling, and plant biomass production to long‐term warming. We find that decomposer organisms are surprisingly resistant to chronic warming, with no responses of bacteria, fungi, or their grazers to temperature (fungivorous nematodes being an exception). Soil organic matter content instead drives spatial variation in microorganism abundances and mineral N availability. The few temperature effects that appear are more focused: root biomass and abundance of root‐feeding nematodes decrease, and nitrification increases with increasing soil temperature. Our results suggest that transient responses of decomposers and soil functioning to warming may stabilize over time following acclimation and/or adaptation, highlighting the need for long‐term, ecosystem‐scale studies that incorporate evolutionary responses to soil warming.

## Graphical abstract

Using a natural soil temperature gradient spanning 10–35°C, we find that soil organisms, soil organic matter content, decomposition, nitrogen cycling, and plant biomass production are surprisingly resistant to chronic warming and that soil organic matter content instead drives the spatial variation in microorganism abundances and mineral N availability. Our results suggest that transient responses of decomposers and soil functioning to warming may stabilize over time following acclimation and/or adaptation, highlighting the need for long‐term, ecosystem‐scale studies that incorporate evolutionary responses to soil warming.

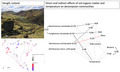

## 1 INTRODUCTION

Terrestrial ecosystems in the Arctic are adapted to harsh abiotic conditions with low soil temperatures and nutrient levels, especially nitrogen (N), and are considered particularly vulnerable to climate change (Overland et al., 2020). As such, soil warming due to climate change could lead to more favorable soil conditions in the Arctic, reducing metabolic constraints on belowground organisms and increasing their activity, which should alter nutrient and carbon (C) dynamics (Nielsen & Wall, 2013). Such changes in the soil environment are intricately linked to aboveground processes as soil communities drive nutrient cycling, and nutrient availability in turn regulates plant and associated above‐ and belowground consumer assemblages (reviewed in Bardgett et al., 2008; Hagedorn et al., 2019; Pugnaire et al., 2019; Wardle et al., 2004).

Ecosystem‐scale impacts of warming are likely to be varied and complex in both space and time, but they need to be disentangled given the potential feedbacks of biogeochemical cycling in the Arctic on climate warming (Chapin et al., 2005, Davidson & Janssens, 2006, see also Conant et al., 2011; Van Gestel et al., 2018). There is also evidence that short‐ and long‐term effects of soil warming may differ, for example as a consequence of adaptation or acclimation (Bradford et al., 2008; Romero‐Olivares et al., 2017), and ecosystems gradually shifting to new equilibria (e.g., Walker et al., 2018). Species‐specific responses also show temporal variation; for example, top‐soil dwelling Collembola in an Icelandic grassland showed opposite responses to short‐ versus long‐term warming (Holmstrup et al., 2018). Moreover, due to lags in the response of interspecific interactions and feedback loops to abiotic change (Alexander et al., 2018; Bardgett et al., 2013), initial observations may provide a misleading impression of ecosystem‐level responses to long‐term climate change. Thus, long‐term studies may be required for warming‐induced changes to be observed (e.g., DeAngelis et al., 2015; Frey et al., 2008; Rinnan et al., 2007).

Due to the high sensitivity of biological processes to temperature, soil warming should accelerate microbial activity (Walker et al., 2018) and amplify the role of soil invertebrates in decomposition processes (Nielsen & Wall, 2013; Wall et al., 2008). However, the response of soil microorganisms to warming and the consequences for soil organic matter (SOM) decomposition appear partly inconsistent (Davidson & Janssens, 2006; Giardina & Ryan, 2000). Changes in soil temperature can also modify the functional role of the microbial community involved in SOM decomposition, for example by shifting from fungal to bacteria‐dominated soil communities (DeAngelis et al., 2015; Hedlund et al., 2004) or vice versa (Yuste et al., 2010; Zhang et al., 2005). Simultaneously, microbial communities are affected by top‐down control on their structure and biomass by soil invertebrates such as nematodes, enchytraeids, and microarthropods (Scheu, 2001; Wardle et al., 2004), and bottom‐up control by plants via organic matter quantity and quality (Conant et al., 2008; Craine et al., 2010; De Deyn et al., 2008; Fierer et al., 2005), and even plant–herbivore interactions (e.g., Rinnan et al., 2009). These food web dynamics and feedback loops may complicate the direct positive effects of increasing temperature on the growth and activity of decomposer organisms, again stressing the importance of long‐term studies that offer time for feedback loops to operate and contribute to the observed effects.

Warming‐induced changes in soil community structure should further impact nutrient cycling, as the trophic structure and predator‐prey interactions in soil food webs are tightly linked to soil functioning (Mikola et al., 2002). A recent meta‐analysis revealed enhanced N mineralization, nitrification and denitrification driven by elevated temperature (Dai et al., 2020). Increased N availability as a consequence of greater net N mineralization rates (Rustad et al., 2001) may in turn lead to heightened N uptake by plants and increased primary production. Changes in soil N availability can also lead to changes in plant biomass allocation and dichotomous responses in above‐ and belowground plant biomass production. Warming tends to increase shoot biomass (Epstein et al., 2012) and nutrient allocation to shoots (DeMarco et al., 2014), at the expense of root biomass (Melillo et al., 2011; Wang et al., 2016; Way & Oren, 2010; although see Sistla et al., 2013; Zamin et al., 2014). Through trophic cascades, such changes in autotroph output can have a bottom‐up effect on plant community structure and the associated epigeal invertebrate assemblage (Bardgett & van der Putten, 2014; Wardle et al., 2004).

Warming typically alters plant phenology and physiology and restructures Arctic plant communities, for example through shrub expansion at the expense of graminoids (Frost & Epstein, 2014; Myers‐Smith et al., 2011; Tape et al., 2006) and bryophytes (Day et al., 2008; Jägerbrand et al., 2009). These changes are likely to alter soil community structure and functioning (e.g., Cramer et al., 2001; Kardol et al., 2010; Wardle et al., 2004) by altering abiotic conditions and the quality (e.g., lignin and N content) and quantity (i.e., litter and plant root exudates) of plant inputs to the soil (Broeckling et al., 2008; Pollierer et al., 2007; Rinnan et al., 2007). In this way, plant community composition actively controls N cycling through a plant–litter–soil–plant feedback loop (Chapman et al., 2006). These impacts may be exacerbated by extended growing seasons as a consequence of climate warming (e.g., Leblans et al., 2017).

Geothermally heated systems act as natural laboratories to provide insight into how soil temperature structures communities and ecosystems (O’Gorman et al., 2014, Sigurdsson et al., 2016). The space‐for‐time substitution facilitates an examination of long‐term responses to warming (i.e. chronic exposure over centennial time‐scales that are likely to incorporate evolutionary responses), which can often differ from short‐term temperature change (Holmstrup et al., 2018; Radujković et al., 2018; Walker et al., 2020). Using a naturally occurring soil temperature gradient in Iceland, we examine the effect of soil temperature on the structure of the soil decomposer community, SOM content and N cycling, and consequently on above‐ and belowground plant and herbivore communities. Soil temperature influences epigeal plant and invertebrate community structure in these systems (Robinson et al., 2018, 2021), but the mechanisms underlying these patterns and the impact of these changes on ecosystem functioning remain unclear. We aim to elucidate the extent to which temperature controls ecosystem functioning through direct and indirect effects on soil microorganisms, invertebrates, and plants. We hypothesize that (H1) increasing soil temperature will sustain higher belowground microbial and invertebrate activity and abundance, promoting SOM decomposition; (H2) greater SOM decomposition will increase N mineralization; and (H3) greater N mineralization, will in turn support higher plant leaf N concentrations and aboveground plant and herbivore biomass.

## 2 METHODS

### 2.1 Study site

**FIGURE 1:**
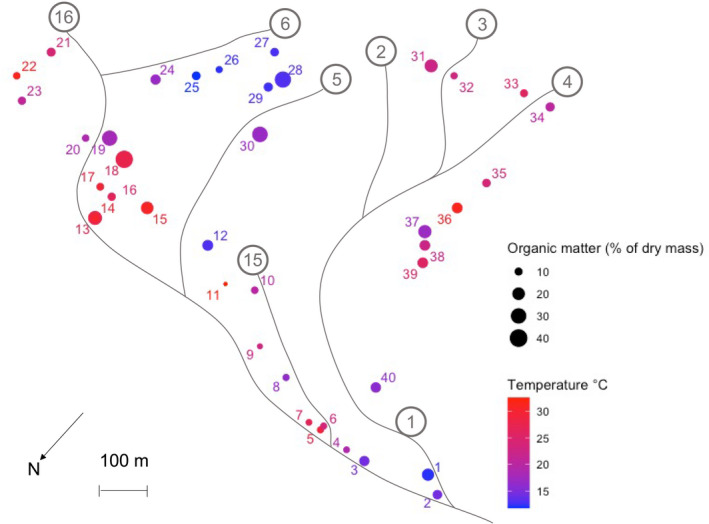
Map showing the distribution, temperature, and soil organic matter content of the 40 sampling locations in the Hengill geothermal valley. Encircled numbers refer to stream codes used in previous publications on the Hengill system (Robinson et al., 2018)

The study was conducted from 15 to 22 August 2018 in the Hengill valley, Iceland (64.03°N 21.18°W). The Hengill valley is characterized by a flat valley floor marked with fumaroles and hot springs, surrounded by slopes of hyaloclastite rocks, along with basalts and acidic rocks (Jousset et al., 2011; Zakharova & Spichak, 2012). The Hengill volcano (one of three main volcanic centers in the area) last erupted 2000 years ago, although the area experiences intense seismicity (Jousset et al., 2011). Due to geothermal heating of the valley, a natural temperature gradient of 10–35°C was present in the soil during sampling (Figure 1). This temperature gradient was found to be consistent over a longer, five‐year sampling period (Figure S1) and over a 2‐month period within a sampling season (see Robinson et al., 2021). Forty plots measuring approximately 1 m^2^ were chosen to evenly span the temperature gradient, enabling us to investigate the links between soil physicochemical properties, vegetation community composition, and above‐ and belowground invertebrate and microbial communities.

### 2.2 Soil properties

Five soil temperature measurements were taken from each plot at 0, 5, and 10 cm soil depth using a Testo 735 thermometer (Testo SE & Co., Germany) attached to a Pt100 sensor (Gräff GmbH, Germany). This was done on three occasions during the sampling period: 15, 18, and 22 August, and a mean temperature for each depth was calculated. Because soil temperatures at all depths were strongly correlated (Figure S2), we use soil temperature at 5 cm depth in all analyses.

For analysis of soil physiochemical properties, three soil cores of 3 cm in diameter were taken from the upper 10 cm soil stratum at each plot; one quarter of each of the three subsamples was pooled for each subsequent analysis, to obtain an estimate per plot. Aboveground plant material, but not roots, was removed from each core. One pooled soil sample was dried in a 70°C drying oven for 24 h, its weight measured before and after drying to determine percentage soil moisture per plot. Twenty grams of the dry soil was then used to measure soil pH by adding 100 ml of distilled water, shaking for 5 min on 150 rpm, letting the sample stand for 2 h, and measuring soil pH from the water layer using an InoLab pH 720 (WTW) probe. Another pooled soil sample was used for analyzing soil mineral P and N concentrations. A 60 g of subsample of fresh soil was extracted in 100 ml of distilled water, filtered through a GF/C (1.2 µm) glass microfiber filter (Whatman, GE Healthcare Europe GmbH) and kept frozen until PO_4_, NH_4_, and NO_3_ concentrations were analyzed using a Lachat QuikChem 8000 analyser (Zallweger Analytics, Inc., Lachat Instruments Division, USA). Total mineral N was calculated as the sum of NH_4_ and NO_3_. Finally, using one of the remaining soil samples, the SOM content was measured as the weight lost from an oven‐dried (105°C for 24 h) soil sample after heating at 550°C for 5 h. Dry root biomass was subtracted from the measure of SOM.

Decomposition rate of soil organic matter was estimated using the Cotton‐strip Assay method (Tiegs et al., 2013). Strips of Fredrix‐brand unprimed 12‐oz. heavyweight cotton fabric (2.5 cm × 8 cm; Style #548) were placed 5 cm below the ground surface at 60 plots concurrently with a Maxim Integrated DS1921G Thermocron iButton temperature logger, on 13 May 2015. Soil temperatures were highly correlated between the studies conducted in 2015 and 2018 (Figure S1; see Robinson et al., 2021 for more details of the 2015 study). Upon collection on 3 July 2015, the strips were rinsed with stream water to remove any residual soil, soaked in 96% ethanol for 30 s to kill bacteria and prevent further decomposition, and dried at 60°C for 12 h. The tensile strength of each cotton strip was measured using a universal testing machine (Instron 5866 with 500 kN tensile holding clamps) and maximum tensile strength recorded. Seven control strips, which had not been placed in the ground, were also tested to provide a baseline tensile strength of the canvas material. % Tensile loss (as a proxy for decomposition) was calculated as (C‐T)/C × 100, where T is the maximum tensile strength recorded for each of the strips from the field, and C is the mean tensile strength of the seven control strips.

### 2.3 Microbial abundance

Bacterial and fungal abundance was estimated from additional soil cores of 3 cm in diameter taken from the upper 4 cm soil stratum (including the litter layer) at each plot. DNA was extracted using the PowerSoil DNA Isolation Kit (Qiagen, Germany). DNA was quantified using the high‐sensitivity Qubit assay (Thermo Fisher Scientific, Switzerland). Relative abundances of bacterial and fungal communities were determined by quantitative PCR (qPCR) on an ABI7500 Fast Real‐Time PCR system (Applied Biosystems, Foster City, CA, USA). PCR amplification of partial bacterial small‐subunit ribosomal RNA genes (region V1–V3 of 16S; primers 27F and 512R) and fungal ribosomal internal transcribed spacers (region ITS2; primers IT3 and ITS4) was performed as described previously (Frey et al., 2020, 2021). For qPCR analyses, 2.5 ng DNA in a total volume of 6.6 µl and 8.4 µl GoTaq qPCRMaster Mix (Promega, Switzerland), containing 1.8 mM of each primer and 0.2 mg ml^−1^ of BSA, were used. The PCR conditions consisted of an initial denaturation at 95ºC for 10 min, 40 cycles of denaturation at 95ºC for 40 s, annealing at 58ºC for 40 s and elongation at 72ºC for 60 s followed by the final data acquisition step at 80ºC for 60 s. The specificity of the amplification products was confirmed by melting‐curve analysis. Three standard curves per target region (correlations ≥0.997) were obtained using 10‐fold serial dilutions (10^−1^ to 10^−9^ copies) of plasmids generated from cloned targets (Frey et al., 2020). Data were converted to represent the average copy number of targets per μg DNA and per g soil.

### 2.4 Vegetation properties

To measure the aboveground biomass (AGB) of vascular plants, the aboveground layer of vegetation was cut and removed from a randomly placed 30 × 30 cm quadrat within each 1 m^2^ plot. The vegetative material was dried in an oven at 70°C for 24 h and weighed to obtain biomass per unit area. AGB was estimated as the biomass of graminoids (including *Carex* spp., *Juncus* spp., *Poa* spp., *Festuca* spp., *Deschampsia* spp.) plus forbs (see Table S3 in Robinson et al., 2018 for the most common species). The total biomass of mosses was also estimated from the same sample. Graminoid leaf N concentration was analyzed from dried and ground leaf material using a LECO CNS‐2000 analyser (LECO Corporation, Saint Joseph, MI, USA).

To estimate the belowground biomass (BGB) of vascular plants, a further soil core of 3 cm in diameter was taken from the 10 cm upper soil stratum (excluding any aboveground plant material from the top layer) at each quadrat established for collecting AGB. While the BGB sampling covered a part of the quadrat only, it still adequately represents the vegetation collected in the AGB sampling; in the soil, plant roots cover a wide horizontal area when searching for nutrients and a soil sample of small area contains a mixture of plant roots growing in a much larger area. The roots were extracted from the soil core by rinsing in water multiple times using a 250‐μm sieve. The roots were then dried at 70°C for 24 h and weighed to obtain biomass per unit area. Root‐to‐shoot ratio was calculated as dry weight of BGB per cm^2^ divided by dry weight of AGB per cm^2^, and the total vascular plant biomass as the sum of AGB and BGB.

### 2.5 Invertebrate community

Three soil cores of 3 cm in diameter were taken from the upper 4 cm soil stratum (including litter layer) at each plot, for analyzing enchytraeid and nematode populations. Enchytraeids were extracted using wet funnels (O’Connor, 1962); one half of each of the three soil cores were pooled for an estimate per plot. Enchytraeids were counted live, classified into size classes (length 0–2, 2.1–4, 4.1–6, 6.1–8, 8.1–10, 10.1–12, or >12 mm) and their biomass was calculated according to Abrahamsen (1973). Wet funnels were also used to extract nematodes (Sohlenius, 1979); one quarter of each of three subsamples was pooled for an estimate per plot. Nematodes were counted live, preserved in 70% ethanol and 50 individuals in each sample were identified and allocated into five trophic groups: bacterivore, fungivore, herbivore, omnivore, and predator (Yeates et al., 1993).

A modified high‐gradient‐extractor (MacFayden, 1961) was used to extract soil micro‐arthropods from soil cores of 5.4 cm in diameter, taken from the upper 4 cm soil stratum (including the litter layer) at each plot. Their total biomass was calculated as the sum of all individual species’ biomasses, obtained using length‐weight regressions (see Robinson et al., 2021). Trophic group (microbivore/detritivore, herbivore, omnivore, predator; Table S1) abundances were also calculated for soil micro‐arthropods.

Aboveground invertebrates were collected using five pitfall traps left for 48 h in each plot. The traps were 7 cm in diameter and 8.5 cm in depth, and were filled with 10 ml of ethylene glycol and 30 ml of stream water to prevent invertebrates escaping and to act as a preservative. Epigeal invertebrate activity density (henceforth “abundance”) was estimated as the total number of individuals found in the five traps, and total biomass as the sum of all individual species’ biomasses. Invertebrates were identified to species level where possible and split into trophic groups (Table S1). Adult Diptera, Hymenoptera, and Lepidoptera were excluded from the data, as they are mainly liquid feeders, parasitoids, or non‐feeding.

### 2.6 Statistical analyses

All statistical analyses were carried out in R, version 4.0.3. A Mantel test (*mantel* function in the *vegan* package) was first used to check for spatial structure in the soil temperature data by comparing pairwise distances between plots (obtained from GPS coordinates using the *earth*.*dist* function in the *fossil* package) with pairwise temperature differences. In line with our hypotheses, we then chose to limit our data analysis to the effects of temperature on SOM, micro‐organisms and fauna directly associated with SOM decomposition and N cycling (bacteria and fungi, bacterivorous and fungivorous nematodes, microbivorous/detritivorous soil micro‐arthropods, and detritivorous enchytraeids), and plants and their herbivores (root‐feeding nematodes and epigeal herbivorous invertebrates). A transformation of log(x) or log(x + 1) was used in all analyses where necessary to meet the assumptions of normality of data distribution, as checked from model residuals.

Tensile loss data were analyzed using a linear mixed effects model (LMEM: *lme* function in the *nlme* package) with temperature as the explanatory variable and plot nested within bank as a random effect to account for potential pseudoreplication (i.e., three plots on the same stream bank; see Robinson et al., 2021 for detailed sampling regime).

Piecewise structural equation modeling (*p*SEM; *psem* function in the *piecewiseSEM* package in R; Lefcheck, 2016) was used to examine the potential causal pathways between variables, and direct and indirect effects of temperature. Although piecewise SEM works with small samples sizes, it is recommended that the ratio of sample size (*n* = 40 in this case) to number of estimated paths is greater than 5 (Grace et al., 2015). For this reason, we could not build a single model incorporating all hypothetical pathways and direct effects of temperature on other variables, but instead built three *a priori* conceptual models to broadly focus on the three ecosystem properties aligned with our hypotheses: SOM content, soil N cycling, and vascular plant biomass production. In the SOM content model, higher temperature is predicted to increase microbial and soil invertebrate biomass, thus leading to lower SOM content (testing H1). In this model, we also include aboveground and belowground vascular plant biomass and moss biomass as predictors for SOM content to allow a plant‐soil feedback, that is, a positive effect of increasing plant production on SOM content. In the N cycling model, temperature, SOM content, micro‐organisms, and microbial‐ and detritus‐feeding invertebrates are predicted to affect soil NH_4_ concentration, and temperature, soil NH_4_ concentration and micro‐organisms to affect soil NO_3_ concentration (testing H2). In the biomass production model, higher temperature and mineral N concentration in the soil are predicted to support higher plant leaf N concentration, higher above‐ and belowground vascular plant biomass, and consequently herbivore biomass (testing H3). Model fit was assessed using Shipley's test of directed separation by way of Fisher's C statistic. Missing pathways (those significant relationships between variables suggested by the test of directed separation) were added to the model and models were compared using Akaike Information Criteria corrected for small sample size (AICc) following removal of nonsignificant pathways (Table S2). Standardized coefficients (β) were recorded for each significant path and indirect effects (β_ind_) were estimated as the product of significant coefficients along the paths.

**FIGURE 2:**
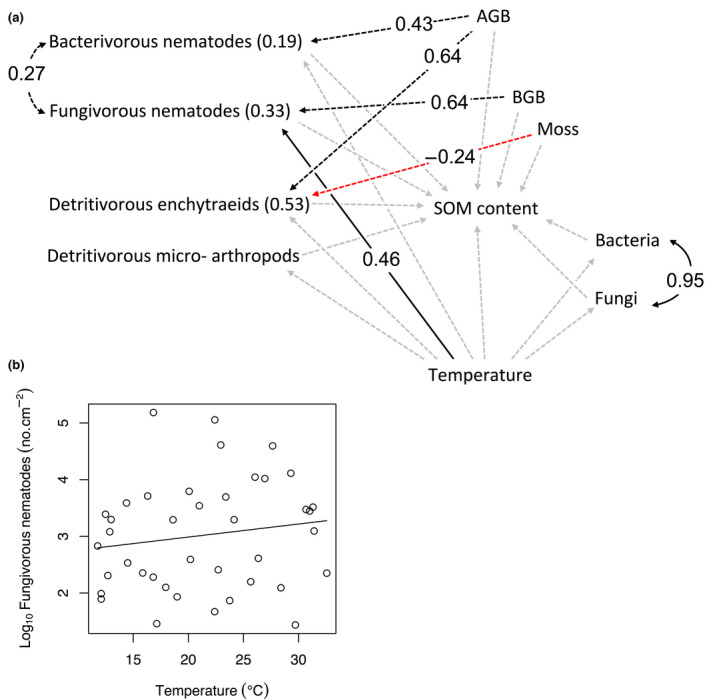
(a) Piecewise structural equation model showing associations between temperature, soil organisms and soil organic matter (SOM) content, and (b) associated significant (*p* < .05) general linear model, showing the direct effect of temperature on fungivorous nematode abundance. Filled black arrows in panel (a) represent significant (*p* < .05) positive hypothesised paths, dashed black and red arrows represent significant positive and negative missing paths, respectively, and dashed grey arrows indicate non‐significant hypothesised paths between variables, as indicated by Shipley's test of directed separation. Standardized coefficients (β) are reported along significant arrows, and *r^2^* values are reported in brackets beside each variable. AGB, aboveground shoot biomass; BGB, belowground root biomass; Moss, dry biomass of mosses. See Figure S3 for a correlation matrix containing associations among all the variables in the *p*SEM model

**FIGURE 3:**
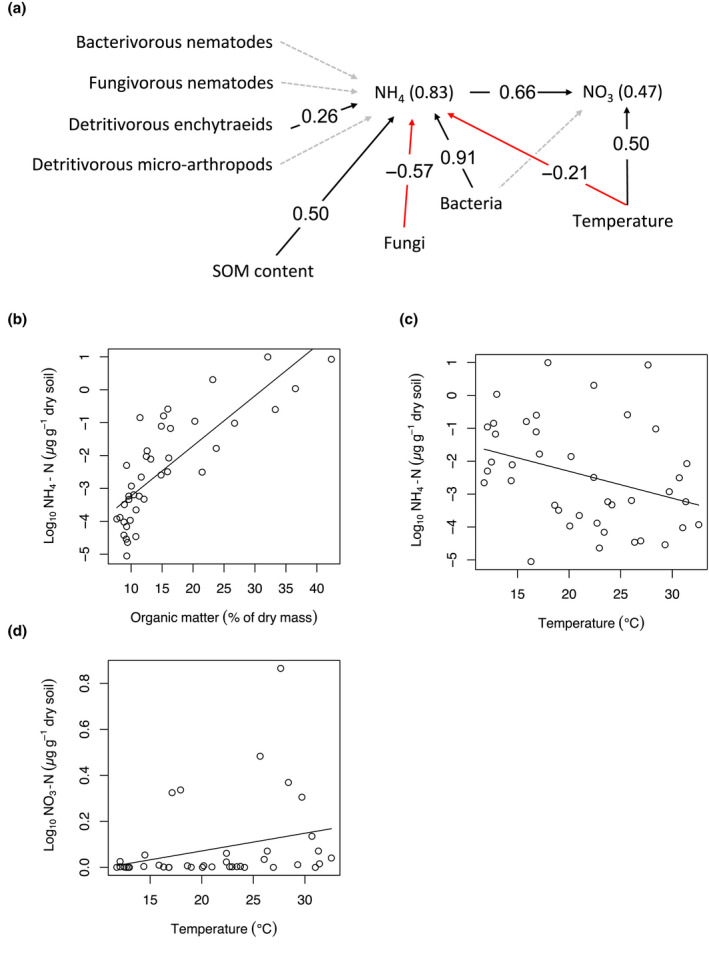
(a) Piecewise structural equation model, and (b–d) associated significant (*p* < .05) general linear models, showing effects of temperature and SOM on soil nitrogen cycling. Filled black and red arrows in panel (a) represent significant (*p* < .05) positive and negative hypothesised paths, respectively, and dashed grey arrows indicate nonsignificant hypothesised paths between variables, as indicated by Shipley's test of directed separation. Standardized coefficients (β) are reported along significant arrows and *r^2^* values are reported in brackets beside each variable. See Figure S4 for a correlation matrix containing associations among all the variables in the *p*SEM model

**FIGURE 4:**
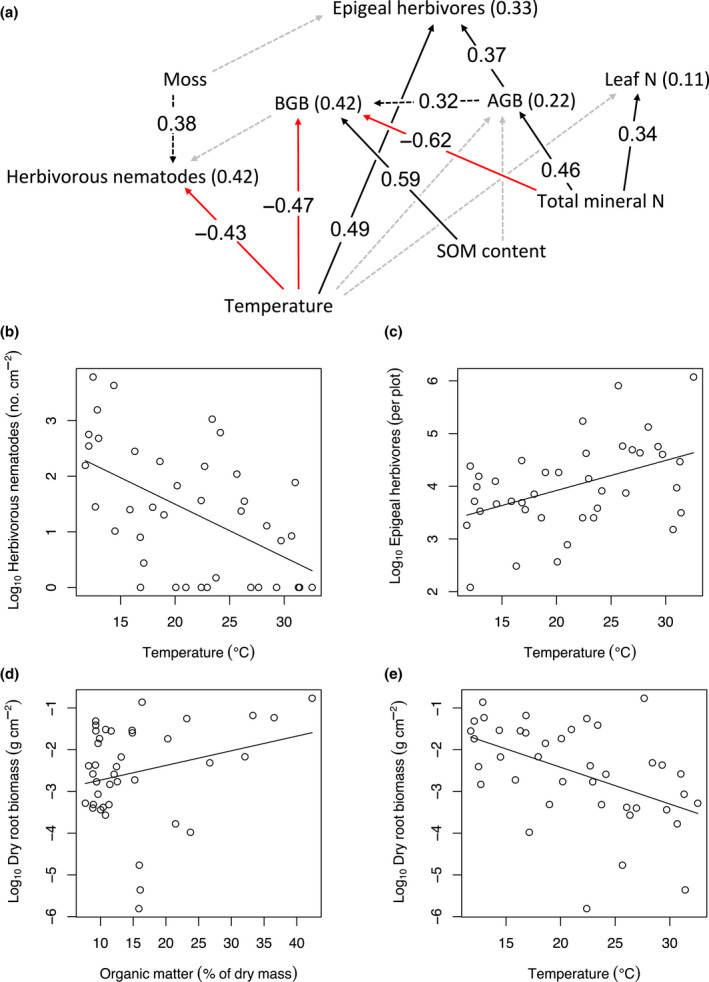
(a) Piecewise structural equation model and (b–e) associated significant (*p* < .05) general linear models, showing effects of temperature and SOM on aboveground biomass production. Filled black and red arrows in panel (a) represent significant (*p* < .05) positive and negative hypothesised paths, respectively, dashed black arrows represent significant positive missing paths, and dashed grey arrows indicate nonsignificant hypothesised paths between variables, as indicated by Shipley's test of directed separation. Standardized coefficients (β) are reported along significant arrows, and *r^2^* values are reported in brackets beside each variable. AGB, aboveground shoot biomass; BGB, belowground root biomass; Moss, dry moss biomass. See Figure S5 for a correlation matrix containing associations among all the variables in the *p*SEM model

Full statistical output of *p*SEM models are presented in Table S3. Associations among all the variables in each *p*SEM model are visually summarized in correlation matrices (Figures S3–S5), while scatterplots in Figures 2, 3, 4 show the statistically significant associations of temperature and SOM with each model's response variables. The statistical significance of direct effects of temperature and SOM content on the response variables shown in these scatterplots was tested using univariate general linear models (GLM; *lm* and *anova* functions), where the effect of one could be tested while controlling for the effect of the other. The GLM results, including estimates of the proportions of the total variance explained by each of the predictors, are shown in full in Table S4.

## 3 RESULTS

### 3.1 Soil temperature, pH, and moisture

There was no significant correlation between pairwise distance and temperature difference between plots (Mantel test: *r* = 0.003; *p* = .413), indicating there was no spatial structure in the temperature data. Soil temperature had no significant effect on soil pH (*F*_(1,38)_ = 2.99, *p* = .092, Figure S6a) or moisture (*F*_(1,38)_ = <0.01, *p* = .990, Figure S6b), but pH decreased (*F*_(1,38)_ = 9.66, *p* = .004, Figure S6d) and soil moisture increased (*F*_(1,38)_ = 129, *p* = <.001, Figure S6e) with increasing SOM content.

### 3.2 SOM content and decomposition rate

In contrast with H1, soil temperature did not influence SOM content (*F*_(1,38)_ = 0.589, *p* = .448; Figure S7). A Cotton‐strip assay study in 2015 further revealed that soil temperature had no effect on the loss of tensile strength of cotton strips, used as a proxy for the general rate of organic matter decomposition (*F*_(1,28)_ = 0.02, *p* = .901; Figure S8).

The first *p*SEM supported these findings, indicating that temperature had no indirect effects on SOM via micro‐organisms or decomposer fauna (bacterial and fungal abundance, bacterivorous nematodes, detritivorous microarthropods, and enchytraeids; Figure 2a). Temperature had a significant positive effect on fungivorous nematodes (Figure 2b), but they did not in turn influence SOM (Figure 2a). Instead, bacterial and fungal abundance and the biomass of detritivorous enchytraeids increased with increasing SOM (Table S4), in contrast to H1, which predicted increasing abundance of microbivorous and detritivorous fauna to decrease SOM. Temperature only explained 0.9, 1.7, and 4.0% in bacterial, fungal, and enchytraeid abundance, whereas SOM content explained 26, 24, and 9.7% of total variation, respectively (Table S4). As a sign of plant–soil feedbacks, the *p*SEM revealed a positive direct effect of aboveground vascular plant biomass on bacterivorous nematodes and detritivorous enchytraeids, a positive direct effect of belowground vascular plant biomass on fungivorous nematodes, and a negative effect of moss biomass on detritivorous enchytraeids (Figure 2a). In contrast, no effects of plant biomass on SOM content appeared in the model (Figure 2a).

### 3.3 Soil N dynamics

The second *p*SEM indicates that SOM controlled N mineralization, but in contrast with H2, higher SOM content was not directly associated with lower, but instead higher NH_4_ concentration and indirectly also higher NO_3_ concentration (β_ind_ = 0.50 × 0.66 = 0.33), and consequently with higher total mineral N (Figure 3a, Table S4). Temperature had a direct positive effect on NO_3_ concentration, but also a direct negative effect on NH_4_ and an indirect negative effect on NO_3_ concentration (β_ind_ = −0.21 × 0.66 = −0.14), leading to no effect of temperature on total soil N (Figure 3a, c, d, Table S4). In contrast with H2, the effects of temperature and SOM were independent of each other and were not linked by the effects of temperature‐induced growth of bacteria, fungi, and decomposer fauna on SOM content (Figures 2 and 3). Accordingly, the second *p*SEM shows that bacteria and enchytraeids had direct positive effects and fungi a direct negative effect on NH_4_, rather than their effects being mediated by SOM content (Figure 3a). Besides higher temperature, NO_3_ concentration was positively influenced by higher NH_4_ concentration (Figure 3a). Spatial variation in SOM content explained a higher percentage of total variation in soil N attributes (40.4–60.7%) than temperature (2.5–11%; Table S4).

### 3.4 Plant and herbivore biomass production

The third *p*SEM showed no direct or indirect effects of temperature on graminoid leaf N concentration and AGB (Figure 4a), in contrast to H3. The positive effect of temperature on epigeal herbivores was also direct and not mediated through leaf N or AGB (Figure 4a). However, higher mineral N concentration in the soil did support higher graminoid leaf N concentrations and higher AGB, and indirectly also higher epigeal herbivore biomass (β_ind_ = 0.46 × 0.37 = 0.17) and higher BGB (β_ind_ = 0.46 × 0.32 = 0.15) (Figure 4a). The effects on AGB can be attributed specifically to graminoid biomass, as forb and moss biomasses were not affected by either SOM or temperature (Table S4). The *p*SEM further revealed that temperature had a direct, negative effect on BGB (Figure 4e; explaining 20.1% of total spatial variation in BGB) and herbivorous nematodes (Figure 4b), and that total mineral N had a direct negative effect on BGB (Figure 4a, Table S4). In the *p*SEM, SOM content had a positive effect on BGB (explaining 16.2% of total variation) (Figure 4a and d, Table S4), and positive effects of SOM also appeared on AGB and graminoid leaf N concentration in univariate tests (explaining 11.3% and 11.1% of total variation, respectively; Table S4).

## 4 DISCUSSION

We show that soil temperature plays a minor role in structuring soil decomposer communities and controlling SOM decomposition, N mineralization and aboveground plant growth, even though previous results from our study system indicate that temperature structures aboveground plant and invertebrate communities (Robinson et al., 2018, 2021). Instead, it appears that SOM content (an indicator of resource availability in soil, which does not vary with temperature), explains a greater proportion of the spatial variation in the structure and functioning of belowground communities (Table S4). SOM and the microbial community drive N mineralization and availability, which in turn supports higher aboveground vascular plant biomass and leaf N concentrations. Temperature has fewer and more focused effects: the abundance of fungivorous nematodes and nitrification rate increase and root mass and root‐feeding nematodes decrease with increasing temperature. These results are in contrast to our expectations based on short‐term (<10 years) temperature manipulations that temperature drives decomposition and N‐mineralization (e.g., Jung et al., 2020; Weedon et al., 2012), and suggest that northern soils and ecosystems, when subjected to a long‐term change in abiotic conditions, have the potential to acclimatize and/or adapt to temperature change. As such, our results corroborate previous findings, which indicate high resistance of High Arctic soil ecosystems to 16 years of experimental warming and fertilization (Lamb et al., 2011), and even >50 years of warming in an Icelandic grassland ecosystem (Radujković et al., 2018).

We hypothesized that decomposition and mineralization would be promoted because of a warming‐induced increase in microbial activity. Surprisingly, however, soil temperature did not affect the rate of decomposition of cotton strips or SOM content at our study site (Figures S7 and S8), despite earlier evidence for high temperature sensitivity of both carbon‐cycle enzymes (Wallenstein et al., 2009) and microbial activity (Koch et al., 2007). The effects of higher temperatures on decomposition may be offset in time by a decrease in the quality of litter with warming (Eliasson et al., 2005; Kirschbaum, 2004), and acclimation of microbial respiration to warming may explain the declining temperature dependence of organic matter decomposition (Bradford, 2013; Davidson & Janssens, 2006). This becomes evident in long‐term studies, as short‐term warming experiments often encompass only the initial substrate depletion phase (Kirschbaum, 2006). For example, a >15 year soil warming experiment in a mid‐latitude, mixed deciduous forest showed that reductions in soil organic C and microbial biomass, in combination with thermal adaptation of microbial respiration, resulted in acclimation of soil respiration (Bradford et al., 2008).

In our study, neither the biomass of primary decomposers (bacteria and fungi) nor the abundance of the majority of animals feeding on them (bacterivorous nematodes and detritivorous enchytraeids and micro‐arthropods) was temperature dependent (Figure 2a, Table S4). These results agree with earlier findings that resource quantity and quality may be more important in determining microbial biomass and activity, than temperature, in high latitude environments (Adamczyk et al., 2020; Frey et al., 2013; Stark, 2007; Weedon et al., 2011). In support of this, we observed a strong positive impact of soil SOM on both bacterial and fungal biomass (Table S4). Results from a meta‐analysis of boreal and subarctic gradient studies suggest that moisture may be more important in determining decomposition rates than temperature alone (Aerts, 2006). At our study site, soil moisture was indeed significantly correlated with SOM content (Figure S6e).

Enchytraeid worms, nematodes, and microarthropods have shown varying responses to warming in previous experiments (Lindo, 2015; Mueller et al., 2016; Sjursen et al., 2005; Thakur et al., 2014, see Aerts, 2006 for review). At our study site, however, the *p*SEM analysis found that the abundance of fungivorous nematodes alone responded to increasing temperature (Figure 2a and b), and even this association disappeared in univariate tests (Table S4). While the most probable explanation for the robustness of most microbial feeders to increasing temperature is the consistent overall biomass of soil microorganisms, it is surprising that neither fungivorous nor bacterivorous nematodes responded to the significant effect of SOM content on fungal and bacterial biomass (Table S4), given that they should readily respond to microbial production in soil (Christensen et al., 1992; Mikola & Setälä, 1998; Sohlenius, 1990). Instead, the *p*SEM suggests that vascular plant shoot biomass drives the trends in bacterivorous nematode and enchytraeid abundance and that root biomass drives the trend in fungivorous nematodes (Figure 2a). This is logical since the growth of microorganisms and soil invertebrates depend on the carbon compounds in root exudate release (Bonkowski et al., 2009; Christensen et al., 1992), which in turn reflects aboveground production of photosynthates, especially in environments where nutrient deficiency limits use of photosynthates for plant growth (Prescott et al., 2020). The negative effect of moss biomass on the biomass of detritivorous enchytraeids could simply be due to moss litter being highly recalcitrant to decomposition (Coulson & Butterfield, 1978; Hobbie, 1996). Altogether, these effects highlight the importance of plant–soil feedbacks for soil functioning and how decomposer communities are under bottom‐up control by plants via organic matter quantity and quality (Broeckling et al., 2008; Craine et al., 2010; De Deyn et al., 2008; Fierer et al., 2005). The reason why abundances of microbial feeders do not follow most of the trends of microbes in our study, could be that total biomass of bacteria and fungi hide trends of subgroups that are most relevant for their feeders.

Although SOM content and decomposition rate (loss of tensile strength of cotton strips) were not affected by soil temperature, we found some signs of temperature effects on bacterial activity, that is, we observed decreasing NH_4_ concentration and increasing NO_3_ concentration with increasing temperature (Figure 3a, c, d). A likely explanation for this trend is that warming elevates the activity of nitrifiers, increasing the conversion of NH_4_ to NO_3_, while not affecting the mineralization of organic N to NH_4_. This finding agrees with earlier studies that have reported increasing nitrification with increasing temperatures in the Arctic (Atkin, 1996; Nadelhoffer et al., 1991; Nadelhoffer & Raich, 1992; Robinson et al., 1995). Simultaneously, higher SOM content promotes N mineralization and indirectly through better NH_4_ availability also nitrification (Figure 3a; Table S4), leading to higher availability of both mineral N forms. Thus, while temperature influences microbial activity and N transformations at our study site, total mineral N availability in the soil seems to depend on SOM content. The significant effect of detritivorous enchytraeid biomass on NH_4_ concentration (Figure 3a) can be explained by the control these organisms exert on N mineralization and assimilation (Mikola et al., 2002).

Higher N mineralization benefits plant growth and plant feeders, as suggested by the direct positive effects of total mineral N on shoot biomass and graminoid leaf N concentration and the indirect positive effects on root mass and epigeal herbivore abundance in our third *p*SEM (Figure 4a). We observed that shoot biomass and graminoid leaf N concentration remain constant along the temperature gradient, which is consistent with the finding that temperature does not affect total mineral N availability but only increases the nitrification rate. Instead, the positive association of SOM content with NH_4_, NO_3_ and total mineral N availability (Table S4) led to a positive effect of SOM content on graminoid leaf N concentration and graminoid shoot mass (Table S4). This is in line with earlier observations that graminoids are the plant group in Arctic environments that most benefit from higher soil nutrient availability (Croll et al., 2005). Higher leaf N concentration could further contribute to faster decomposition and higher soil mineral N availability (Mikola et al., 2018; Parton et al., 2007), thus leading to a positive plant–soil feedback loop. Whether inorganic N is available as NH_4_ or NO_3_ may have significance in plant community assembly because species differ in their preferred source of N (Britto & Kronzucker, 2013). In the Arctic, plants are typically adapted to use NH_4_ or organic N like amino acids, but not NO_3_, because it is rarely available (Kielland, 1994). Increasing NO_3_ concentrations with warming might thus partly explain the decreasing plant species α‐diversity and richness, and greater species turnover with warming previously observed in Hengill (Robinson et al., 2018).

Although we did not find temperature to have a direct effect on vascular plant shoot biomass, or indirect effects mediated through N availability, we found that increasing soil temperature had a negative effect on belowground root biomass, leading to a decrease in the root to shoot ratio (Figure 4a). This finding is in line with previous research, which also indicates that warming shifts biomass allocation away from roots (Melillo et al., 2011; Pregitzer et al., 2000; Wang et al., 2016). Decreasing root biomass would be a logical response if increasing temperatures led to greater nutrient availability. The direct negative effect of total mineral N on root biomass that was revealed by the third *p*SEM (Figure 4a) is a manifestation of this effect. However, this was not the case in our study: warming did not affect mineral N (Figure 4a) and led to lower mineral P concentrations (Figure S6c). It is therefore unlikely that the explanation for decreasing root biomass is a greater availability of nutrients with warming. Instead, the explanation may be related to plant physiology; for example, root function may be more efficient in warmer environments, especially considering root processes are temperature dependent (Kaspar & Bland, 1992), so fewer roots are required to maintain AGB. We also observed fewer herbivorous nematodes with increasing temperature (Figure 4a and b), which most likely is due to the concordant reduction in root biomass although the indirect effect of temperature on root feeders through root biomass was not supported by the *p*SEM (Figure 4a). Unlike in the case of root feeding nematodes, the positive effect of temperature on epigeal invertebrate herbivores (Figure 4a and c) was apparently not mediated by changes in plant growth since plant shoot mass and leaf N concentration did not respond to temperature (Figure 4a). As epigeal invertebrates are able to move freely over large distances, they may be more readily able to choose their preferred thermal habitat compared with soil dwelling animals (see Mod et al., 2020), whose movement is more restricted within the soil.

## 5 CONCLUSION

Although short‐term warming treatments have reported significant effects of warming on soil microbial biomass, N mineralization (see Davidson & Janssen, 2006; Kirschbaum, 2006 for reviews), and consequently on aboveground plant production (e.g., Silfver et al., 2020), we did not find those effects along our natural soil temperature gradient. Instead, spatial variation in many soil attributes was driven by SOM content, and temperature had more focused effects, for instance on nitrifying bacteria and root biomass and root‐feeding nematodes. This suggests that long‐term warming, over the decadal time scale of climate change, may allow for stabilization of early acute responses to increasing temperature, and subsequent acclimation and adaptation of decomposer organisms that mostly have short life cycles. Thus, the initial effects of a warming climate on the structure of soil decomposer communities, N mineralization, and aboveground plant growth may wane, such that ecosystem functioning is later, for the most part, governed by baseline resource availability.

## AUTHOR CONTRIBUTION

S.I.R., J.M., and E.J.O.G. designed the research; S.I.R. and E.J.O.G. collected the data; S.I.R., B.F., and M.H. processed the data; S.I.R. analyzed the data; S.I.R. and J.M. led the writing of the manuscript. All the authors contributed to the drafts and gave final approval for publication.

## Supporting information

Supplementary Material

## Data Availability

The data that support the findings of this study are openly available in Dryad at https://doi.org/10.5061/dryad.rxwdbrvbd.
