# Supplementary material for: Soil organic matter, rather than temperature, determines the structure and functioning of subarctic decomposer communities
Source: Glob Chang Biol. 2022 Mar 21;28(12):3929–43. doi: 10.1111/gcb.16158 (PMC9310844; doi:10.1111/gcb.16158)
Supplement: Supplementary file 1 — Supplementary Material [file GCB-28-3929-s001.docx]

**Supporting Information: Soil organic matter, rather than temperature, determines the structure and functioning of subarctic decomposer communities**

Sinikka I. Robinson^1*^, Eoin J. O’Gorman^2^, Beat Frey^3^, Marleena Hagner^1,4^, Juha Mikola^1,5^

*^1^ Ecosystems and Environment Research Programme, University of Helsinki, Niemenkatu 73, 15140 Lahti, Finland*

*^2^ School of Life Sciences, University of Essex, Wivenhoe Park, Colchester, CO4 3SQ, UK*

*^3^ Swiss Federal Research Institute WSL, 8903 Birmensdorf, Switzerland*

*^4^ Natural Resources Institute Finland (Luke), 31600 Jokioinen, Finland*

*^5^ Natural Resources Institute Finland (Luke), Latokartanonkaari 9, 00790 Helsinki, Finland*

*** *Correspondence to: sinikka.robinson@helsinki.fi*

**Figure S1.** Pearson correlations between soil spot temperatures taken across five years of sampling in the Hengill valley. Years 2012-2014 include measurements from 96 plots (see Robinson et al. 2018 for detailed sampling regime and plot locations), 2015 includes data from 50 of these same plots (see Robinson et al. 2021), and 2016 includes data from 30 of these same plots.


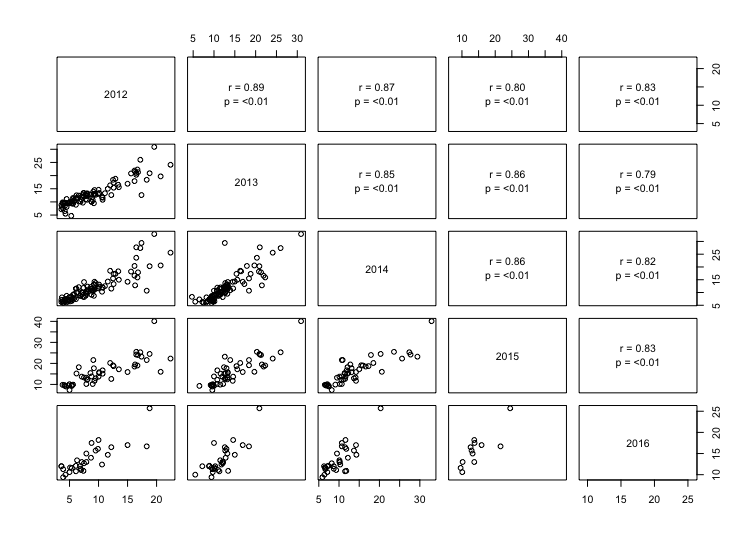


**Figure S2.** Pearson correlations between soil temperatures (°C) at 0, 5, and 10 cm depth, taken on three occasions during the sampling period: 15^th^, 18^th^, and 22^nd^ August 2018.


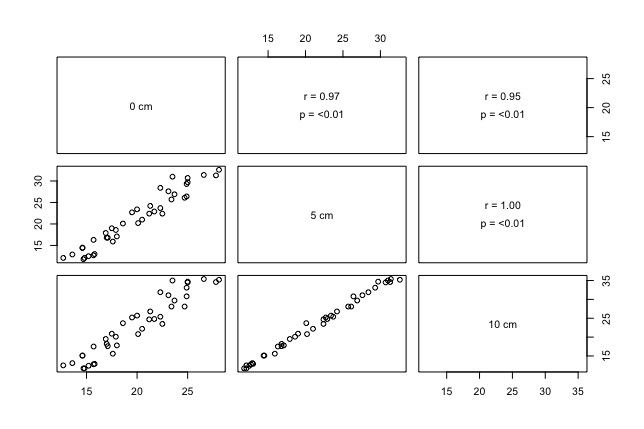


**Figure S3.** Pearson correlations between all variables included in the soil organic matter (SOM) decomposition piecewise structural equation model (*p*SEM; Table S3), illustrated in Fig. 2. Abbreviations for variables are defined in Table S2.

**Figure S4.** Pearson correlations between all variables included in the soil nitrogen (N) cycling *p*SEM (Table S3), illustrated in Fig. 3. Abbreviations for variables are defined in Table S2.

**Figure S5.** Pearson correlations between all variables included in the vascular plant biomass production *p*SEM (Table S3), illustrated in Fig. 4. Abbreviations for variables are defined in Table S2.

**Figure S6.** Effect of soil temperature at 5 cm belowground and SOM content on soil pH, moisture, and phosphate (PO_4_) concentrations. Solid lines indicate a significant (*p*<0.05) linear relationship as tested using a linear regression.

**
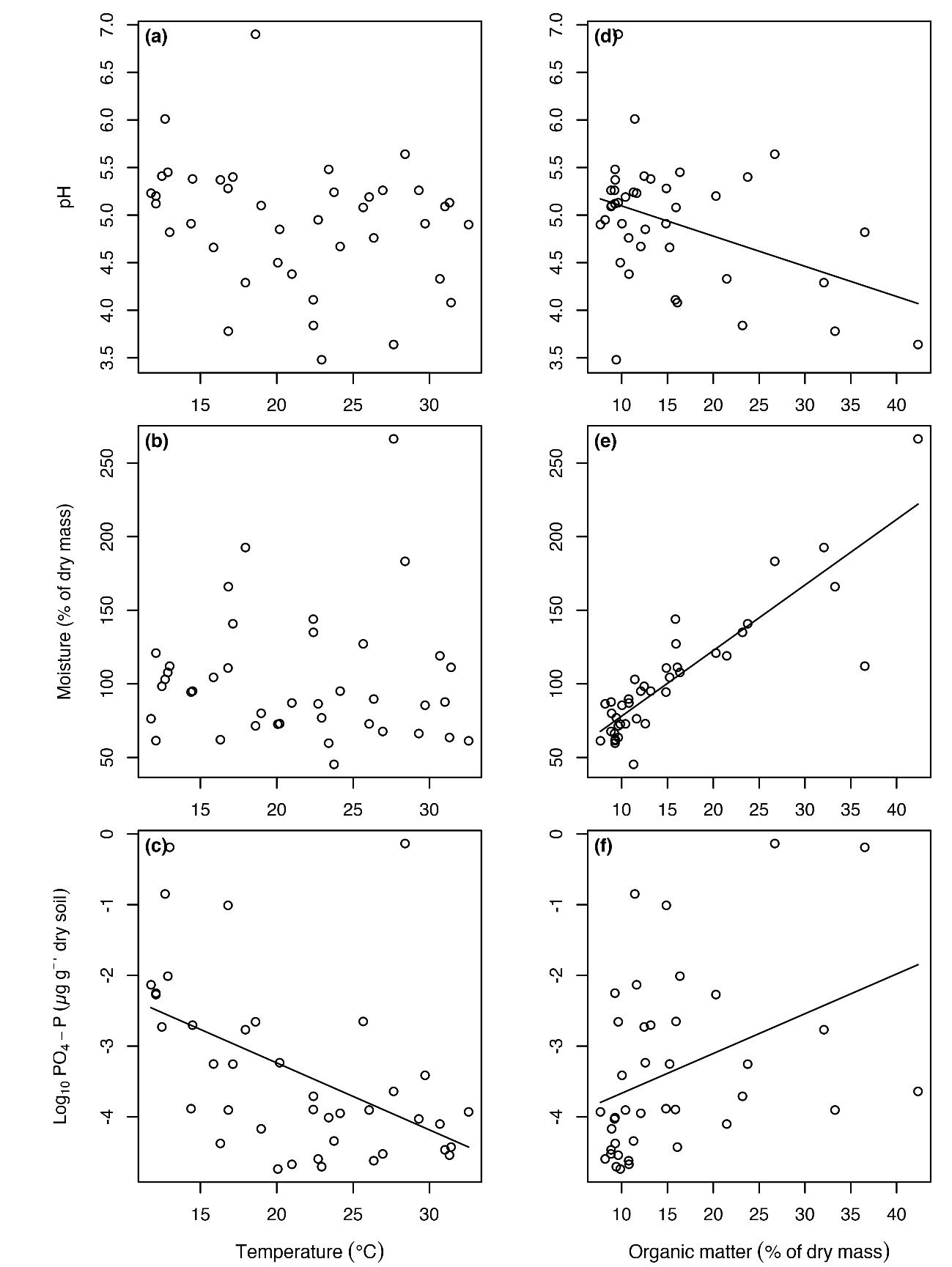
**

**Figure S7.** Effect of soil temperature at 5 cm depth on soil organic matter content (excluding root biomass).


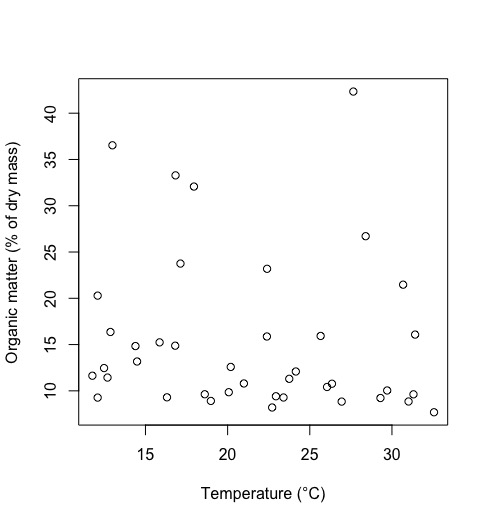


**Figure S8.** Effect of soil temperature at 5 cm below-ground on percentage loss of tensile strength of cotton canvas strips (used as a proxy for decomposition). The data was collected during sampling in May – July 2015 (see Robinson et al. 2021 for a more detailed sampling regime).


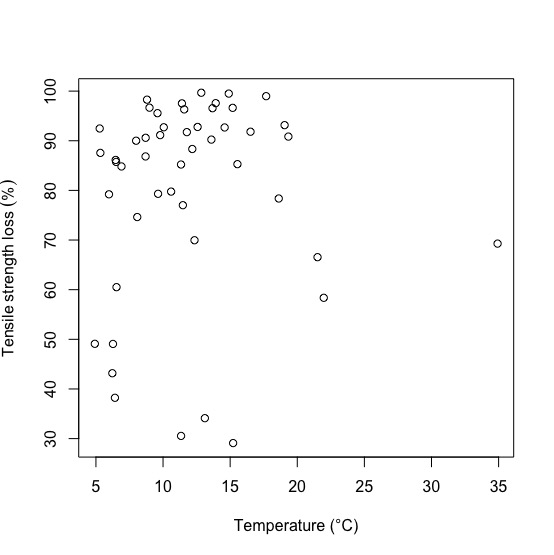


**Table S1.** List of soil and epigeal arthropod taxa, their feeding group and associated references, and their total abundance across all plots. Arthropods were sampled using pitfall traps and soil cores at 40 plots across the soil temperature gradient.

| **Taxon** | **Feeding group** | **Reference** | **Abundance in pitfall traps** | **Abundance in soil cores** |
| --- | --- | --- | --- | --- |
| Acari non-oribatid^[[1]](#footnote-1)^ | Omnivore | 3, 8, 11, 23, 30 | 569 |  |
| Acari oribatid | Microbivore/  Detritivore | 3, 8, 11, 20, 37 | 170 | 332 |
| *Amara quenseli* | Omnivore | 5 | 1 |  |
| Aphididae | Herbivore | 8, 23 | 191 |  |
| Aphididae nymph | Herbivore | 8 | 1 |  |
| *Arctorthezia cataphracta* | Herbivore | 8, 11, 15 | 2 |  |
| *Arctosa alpigena* | Predator | 8, 23, 30 (based on *A. leopardus*) | 1 |  |
| *Arctosa lutetiana* | Predator | 8, 10, 23, 30 (based on *A. leopardus*) | 4 |  |
| *Arion* sp. | Omnivore | 10 | 2 |  |
| *Bembidion bipunctatum* | Predator | 5 (based on *B. hastii*), 8, 28, 30, 38 | 1 |  |
| *Bembidion femoratum* | Predator | 5 (based on *B. hastii*), 8, 23, 30, 38 | 1 |  |
| *Calathus melanocephalis* | Predator | 8, 23, 30, 38, 39 | 4 |  |
| Cicadellidae sp. | Herbivore | 11, 23 | 5 |  |
| *Cochlicopa lubrica* | Omnivore | 32 | 1 |  |
| Coleoptera larva | Predator | 8, 12, 29 | 5 |  |
| *Damaeus* sp. | Microbivore/  Detritivore | 20 | 25 |  |
| Delphacidae sp. | Herbivore | 23 | 38 |  |
| *Deroceras* sp. | Omnivore | 2, 6 | 39 |  |
| Diptera larva | Omnivore | 8 | 1 |  |
| Entomobryomorpha | Microbivore/  Detritivore | 8, 11, 23, 26 | 1414 | 660 |
| Gonatium | Predator | 1, 8, 11 | 3 |  |
| Hemiptera nymph | Herbivore | 8 | 579 |  |
| *Hypnoidus riparius* | Herbivore | 7 | 8 |  |
| *Laemostenus complanatus* | Predator | 8, 30 | 2 |  |
| Lepidoptera larva | Herbivore | 9, 24 | 1 |  |
| Linyphiidae | Predator | 5, 8, 11, 23, 30 | 25 |  |
| Lycosidae | Predator | 8, 11, 14, 23, 30, 35 (based on *P. lugubris*) | 11 |  |
| Lycosidae juvenile | Predator | 8, 30, 35 (based on *P. lugubris*) | 422 |  |
| Mesostigmata | Predator | 8 |  | 277 |
| Miridae sp. | Omnivore | 18, 23 | 2 |  |
| *Mitopus morio* | Predator | 11, 19, 30, 34, 36 | 69 |  |
| Neelipleona | Microbivore/  Detritivore | 11, 23, 26 |  | 14 |
| *Notiophilus biguttatus* | Predator | 8, 16, 17, 23 | 2 |  |
| *Pardosa agrestis* | Predator | 8, 11, 23, 33 | 2 |  |
| *Pardosa palustris* | Predator | 8, 11, 13, 23, 33 | 83 |  |
| *Pardosa prativaga* | Predator | 8, 11, 23, 25 | 23 |  |
| *Patrobus septentrionis* | Predator | 8, 14, 23, 30 | 1 |  |
| *Pirata piraticus* | Predator | 8, 11, 13, 23 | 418 |  |
| *Piratula hygrophila* | Predator | 8, 11, 23 | 70 |  |
| Poduromorpha | Microbivore/  Detritivore | 8, 11, 23 | 993 | 118 |
| *Porhomma montanum* | Predator | 1, 8, 11, 23, 30 | 9 |  |
| Prostigmata | Omnivore | 8 |  | 45 |
| Psyllidae sp. | Herbivore | 22 | 13 |  |
| *Pterostichus adstrictus* | Predator | 4 (based on *P. melanarius*), 8, 14, 23, 30, 38 | 2 |  |
| *Pterostichus diligens* | Predator | 4 (based on *P. melanarius*), 8, 14, 21, 23, 30, 38 | 51 |  |
| *Pterostichus nigrita* | Predator | 4 (based on *P. melanarius*), 8, 14, 23, 30, 38 | 135 |  |
| *Pterostichus strenuus* | Predator | 4 (based on *P. melanarius*), 8, 14, 23, 30, 38 | 4 |  |
| Staphylinidae | Predator | 8, 11, 23, 27, 30 | 9 |  |
| Symphypleona | Herbivore | 8, 26 | 2274 | 13 |
| Thysanoptera | Herbivore | 8, 23 | 21 |  |
| *Trechus obtusus* | Predator | 31, 38 | 58 |  |

**References**

1. Agustí N, Shayler SP, Harwood JD, Vaughan IP, Sunderland K, Symondson W. (2003) Collembola as alternative prey sustaining spiders in arable ecosystems: prey detection within predators using molecular markers. Molecular Ecology 12: 3467-3475.
2. Barrada M, Iglesias J, Castillejo J. (2004) Utilization of weeds and crop plants by the pest slug, Deroceras reticulatum (Müller, 1774). Biological agriculture & horticulture 22: 185-98.
3. Bartrons M, Gratton C, Spiesman BJ, Vander Zanden MJ. (2015) Taking the trophic bypass: aquatic‐terrestrial linkage reduces methylmercury in a terrestrial food web. Ecological Applications 25: 151-9.
4. Bohan DA, Bohan AC, Glen DM, Symondson WOC, Wiltshire CW, Hughes L. (2000) Spatial dynamics of predation by carabid beetles on slugs. Journal of Animal Ecology 69, 367–379.
5. Bråten AT, Flø D, Hågvar S, Hanssen O, Mong CE, Aakra K. (2012) Primary succession of surface active beetles and spiders in an alpine glacier foreland, central south Norway. Arctic, Antarctic, and Alpine Research 44: 2-15.
6. Cook RT, Bailey SE, McCrohan CR, Nash B, Woodhouse RM. (2000) The influence of nutritional status on the feeding behaviour of the field slug, Deroceras reticulatum (Müller). Animal Behaviour 59: 167-76.
7. Coope GR, Böcher J. (2000) Coleoptera from the Late Weichselian deposits at Nørre Lyngby, Denmark and their bearing on palaeoecology, biogeography and palaeoclimate. Boreas 29: 26-34.
8. Crotty FV, Blackshaw RP, Inger R, Murray PJ. (2013) Divergence of feeding channels within the soil food web determined by ecosystem type. Ecology and Evolution 4: 1-3.
9. Danell K, Ericson L. (1990) Dynamic relations between the antler moth and meadow vegetation in northern Sweden. Ecology 71: 1068-77.
10. Dolejš P, Kubcová L, Buchar J. (2008) Subterrestrial life of Arctosa lutetiana (Araneae, Lycosidae). The Journal of Arachnology 36: 202-3.
11. Dreyer J, Hoekman D, Gratton C. (2012) Lake‐derived midges increase abundance of shoreline terrestrial arthropods via multiple trophic pathways. Oikos 121: 252-8.
12. Egert M, Wagner B, Lemke T, Brune A, Friedrich MW. (2003) Microbial community structure in midgut and hindgut of the humus-feeding larva of Pachnoda ephippiata (Coleoptera: Scarabaeidae). Applied and Environmental Microbiology 69: 6659-68.
13. Eitzinger B, Roslin T, Vesterinen E, Robinson SI, O'Gorman EJ. (2021) Temperature affects both the Grinnellian and Eltonian dimensions of ecological niches–a tale of two Arctic wolf spiders. Basic and Applied Ecology
14. Ekschmitt K, Weber M, Wolters V (1997) Spiders, Carabids, and Staphylinids: The ecological potential of predatory macroarthropods. In: Benckiser G (Ed.), Fauna in Soil Ecosystems. Marcel Dekker, New York, pp. 307-362.
15. Ericson L, Wenneström A (1997) The effect of herbivory on the interaction between the clonal plant *Trientalis europaea* and its smut fungus *Urocystis trientalis*. Oikos 80: 107-111.
16. Ernsting G, Isaaks JA, Berg MP. (1992) Life cycle and food availability indices in Notiophilus biguttatus (Coleoptera, Carabidae). Ecological Entomology 17: 33-42.
17. Ernsting G. (1977) Effects of food deprivation and type of prey on predation by Notiophilus biguttatus F.(Carabidae) on springtails (Collembola). Oecologia 31: 13-20.
18. Gillespie DR (2017) Omnivory – A Flexible Feeding Strategy. In: Reference Module in Life Sciences, Elsevier. <https://doi.org/10.1016/B978-0-12-809633-8.12260-5>
19. Hartmann T, Häggström H, Theuring C, Lindigkeit R, Rahier M. (2003) Detoxification of pyrrolizidine alkaloids by the harvestman Mitopus morio (Phalangidae) a predator of alkaloid defended leaf beetles. Chemoecology 13: 123-7.
20. Hayes AJ. (1963) Studies on the feeding preferences of some phthiracarid mites (Acari: Oribatidae). Entomologia experimentalis et applicata 6: 241-56.
21. Hengeveld R. (1979) Qualitative and quantitative aspects of the food of ground beetles (Coleoptera, Carabidae): a review. Netherlands Journal of Zoology 30: 555-63.
22. Hodkinson ID, Bird JM. (2015) Psyllidae (Jumping plant-lice, psyllids). The Greenland Entomofauna: An Identification Manual of Insects, Spiders and their Allies 10: 113.
23. Hoekman D, McCary MA, Dreyer J, Gratton C. (2019) Reducing allochthonous resources in a subarctic grassland alters arthropod food webs via predator diet and density. Ecosphere 10: e02593.
24. Hunter MD, Kozlov MV, Itämies J, Pulliainen E, Bäck J, Kyrö EM, Niemelä P. (2014) Current temporal trends in moth abundance are counter to predicted effects of climate change in an assemblage of subarctic forest moths. Global change biology 20: 1723-37.
25. Jespersen LB, Toft S. (2003) Compensatory growth following early nutritional stress in the wolf spider Pardosa prativaga. Functional Ecology 1: 737-46.
26. König T, Kaufmann R, Scheu S. (2011) The formation of terrestrial food webs in glacier foreland: evidence for the pivotal role of decomposer prey and intraguild predation. Pedobiologia 54: 147-52.
27. Leschen RA, Newton AF. (2003) Larval description, adult feeding behavior, and phylogenetic placement of Megalopinus (Coleoptera: Staphylinidae). The Coleopterists Bulletin 57: 469-93.
28. Lovei GL, Sunderland KD. (1996) Ecology and behavior of ground beetles (Coleoptera: Carabidae). Annual review of entomology 41: 231-56.
29. Luff ML. (1974) Adult and larval feeding habits of Pterostichus madidus (F.)(Coleoptera: Carabidae). Journal of Natural History 8: 403-9.
30. Mellbrand K, Hambäck PA. (2010) Coastal niches for terrestrial predators: a stable isotope study. Canadian Journal of Zoology 88: 1077-85.
31. Mitchell B. (1963) Ecology of two carabid beetles, Bembidion lampros (Herbst) and Trechus quadristriatus (Schrank). The Journal of Animal Ecology 1:377-92.
32. Nordsieck., Robert. "Pillar Snails (Cochlicopidae)". <http://www.molluscs.at/gastropoda/terrestrial.html?/gastropoda/terrestrial/cochlicopidae.html> Retrieved 29 April 2021.
33. Nyffeler M, Benz G. (1988) Feeding ecology and predatory importance of wolf spiders (Pardosa spp.)(Araneae, Lycosidae) in winter wheat fields 1. Journal of Applied Entomology 106: 123-34.
34. Nyffeler M, Symondson WO. (2001) Spiders and harvestmen as gastropod predators. Ecological Entomology 26: 617-28.
35. Oelbermann K, Scheu S. (2002) Effects of prey type and mixed diets on survival, growth and development of a generalist predator, Pardosa lugubris (Araneae: Lycosidae). Basic and Applied Ecology 3: 285-91.
36. Phillipson JA. (1960) contribution to the feeding biology of Mitopus morio (F)(Phalangida). The Journal of Animal Ecology 1:35-43.
37. Schneider K, Renker C, Maraun M (2005) Oribatid mite (Acari, Oribatida) feeding on ectomycorrhizal fungi. Mycorrhiza 16: 67-72.
38. Vanbergen AJ, Woodcock BA, Koivula M, Niemela J, Kotze JD, Bolger T, Golden V, Dubs F, Boulanger G, Serrano J, Lencina JL, Serrano A, Aguiar C, Grandchamp A, Stofer S, Szel G, Ivits E, Adler P, Markus J, Watt AD (2010) Trophic level modulates carabid beetle responses to habitat and landscape structure: a pan-European study. Ecological Entomology 35: 226-235.
39. Vucic‐Pestic O, Rall BC, Kalinkat G, Brose U. (2010) Allometric functional response model: body masses constrain interaction strengths. Journal of Animal Ecology 79: 249-56.

**Table S2.** Piecewise structural equation model selection using corrected Akaike Information Criterion corrected for small sample size (AICc) and Shipley’s test of directed separation. To evaluate model fit, we used Fisher’s C statistic and its associated *p*-value for each model (*p* > 0.05 indicates adequate model fit). Missing pathways (where *p* < 0.05) were added to the model, and subsequently all non-significant pathways were removed from the model. AICc values were compared to obtain the best fitting model (lowest AICc value with ΔAICc > 2). Variables: T = temperature; SOM = soil organic matter content; NH_4_ = soil ammonium concentration; NO_3_ = soil nitrate concentration; B = bacterial abundance; F = fungal abundance; DM-A = detritivorous micro-arthropod abundance; DE = detritivorous enchytraeid biomass; BN = bacterivorous nematode abundance; FN = fungivorous nematode abundance; HN = herbivorous, or root-feeding nematode abundance; AGB = aboveground vascular plant biomass; BGB = root biomass; MB = moss biomass; LN = graminoid leaf nitrogen content; EH = epigeal herbivore abundance; TN = total mineral nitrogen.

|  | AICc | Fisher’s C | *p* |
| --- | --- | --- | --- |
| **Decomposition Model** |  |  |  |
| *Hypothetical model* | 911.484 | 145.08 | 0 |
| B %~~% F  B ~ T  F ~ T  DM-A ~ T  DE ~ T  BN ~ T  FN ~ T  SOM ~ T + B + F + DM-A + DE + BN + FN + AGB + BGB + MB |  |  |  |
| *Model including missing pathways* | 1136.58 | 43.66 | 0.788 |
| B %~~% F  FN %~~% BN  B ~ T  F ~ T  DM-A ~ T  DE ~ T + AGB + BGB + MB  BN ~ T + AGB  FN ~ T + BGB  SOM ~ T + B + F + DM-A + DE + BN + FN + AGB + BGB + MB |  |  |  |
| *Model including missing pathways & excluding non-significant pathways* | 53.924 | 15.75 | 0.61 |
| B %~~% F  FN ~ BN  DE ~ AGB + MB  BN ~ AGB  FN ~ T + BGB |  |  |  |
|  |  |  |  |
| **N cycling model** |  |  |  |
| *Hypothetical model* | 77.36 | 16.42 | 0.173 |
| NH4 ~ T + SOM + B + F + DM-A + DE + BN + FN  NO3 ~ T + NH_4_ + B |  |  |  |
| *Model excluding non-significant pathways* | 46.39 | 10.48 | 0.23 |
| NH_4_ ~ T + SOM + B + DE  NO_3_ ~ T + NH_4_ |  |  |  |
|  |  |  |  |
| **Biomass production model** |  |  |  |
| *Hypothetical model* | 243.26 | 51.30 | 0.029 |
| AGB ~ T + SOM + TN  BGB ~ T + SOM + TN  LN ~ T + TN  EH ~ T + AGB + MB  HN ~ T + BGB |  |  |  |
| *Model including missing pathways* | 228.53 | 29.99 | 0.466 |
| AGB ~ T + SOM + TN  BGB ~ T + SOM + TN + AGB  LN ~ T + TN  EH ~ T + AGB + MB  HN ~ T + BGB + MB |  |  |  |
| *Model including missing pathways & excluding non-significant pathways* | 164.72 | 38.24 | 0.55 |
| AGB ~ TN  BGB ~ T + SOM + TN + AGB  LN ~ TN  EH ~ T + AGB  HN ~ T + MB |  |  |  |

**Table S3.** Best-fit piecewise structural equation models (as selected using corrected Akaike information criterion corrected for small sample size and Shipley’s test of directed separation) for decomposition, nitrogen cycling, and biomass production. Both standardised and unstandardised coefficients are reported. Only significant (*p* < 0.05) pathways were included in the models.

| Model | Path | Estimate | Crit. value | *p* | Std. Estimate |
| --- | --- | --- | --- | --- | --- |
| Decomposition | B %~~% F  FN ~ BN | 0.95  0.27 | 18.28  1.70 | <0.001  0.049 | 0.95  0.27 |
|  | DE ~ AGB | 1.42 | 5.62 | <0.001 | 0.64 |
|  | DE ~ MB | -3.42 | -2.14 | 0.039 | -0.24 |
|  | BN ~ AGB | 0.68 | 2.95 | 0.005 | 0.43 |
|  | FN ~ T | 0.07 | 3.01 | 0.005 | 0.46 |
|  | FN ~ BGB | 0.52 | 4.13 | <0.001 | 0.64 |
|  |  |  |  |  |  |
| N cycling | NH_4_ ~ T  NH_4_ ~ SOM | -0.05  0.15 | -3.22  6.42 | 0.003  <0.001 | -0.21  0.50 |
|  | NH_4_ ~ B  NH_4_ ~ F | 0.58  -0.35 | 4.49  -2.85 | 0.001  0.007 | 0.91  -0.57 |
|  | NH_4_ ~ DE | 0.30 | 3.78 | 0.001 | 0.30 |
|  | NO_3_ ~ T  NO_3_ ~ SOM | 0.01  0.07 | 3.95  5.16 | <0.001  <0.001 | 0.50  0.66 |
|  |  |  |  |  |  |
| Biomass production | AGB ~ TN | 0.18 | 3.24 | 0.003 | 0.47 |
|  | BGB ~ T  BGB ~ SOM  BGB ~ TN | -0.09  0.08  -0.46 | -3.63  2.79  -2.72 | 0.001  0.009  0.010 | -0.47  0.59  -0.62 |
|  | BGB ~ AGB  LN ~ TN | 0.62  0.04 | 2.21  2.19 | 0.033  0.034 | 0.32  0.34 |
|  | EH ~ T | 0.06 | 3.61 | 0.001 | 0.49 |
|  | EH ~ AGB | 0.50 | 2.76 | 0.009 | 0.37 |
|  | HN ~ T | -0.08 | -3.32 | 0.002 | -0.43 |
|  | HN ~ MB | 4.52 | 2.94 | 0.006 | 0.38 |

**Table S4.** Effects of soil temperature at 5 cm depth and soil organic matter (SOM) content (excluding root biomass) on mineral N concentrations, soil micro-organisms, invertebrate fauna, and plant properties, tested using univariate General Linear Models. *F* and *p* indicate *F*-statistics and *p*-values, respectively (*p* < 0.05 shown in **bold**), while %SS indicates the percentage of total sums of squares, i.e. the proportion of variance explained by the explanatory variable. Asterisks indicate Log_10_-transformation of the response variable for the analysis.

|  | Temperature (°C) | | | SOM (% of dry mass) | | |
| --- | --- | --- | --- | --- | --- | --- |
|  | *F* | *p* | %SS | *F* | *p* | %SS |
| *Micro-organisms* |  |  |  |  |  |  |
| Bacterial abundance^*^ | 0.43 | 0.517 | 0.85 | 13.2 | **<0.001** | 26.1 |
| Fungal abundance^*^ | 0.86 | 0.360 | 1.73 | 11.8 | **0.001** | 23.7 |
| Bacterial:fungal ratio^*^ | 0.69 | 0.411 | 1.84 | 0.003 | 0.955 | 0.01 |
| *Invertebrates* |  |  |  |  |  |  |
| Detritivorous enchytraeid biomass^*^ | 1.70 | 0.201 | 3.96 | 4.17 | **0.048** | 9.72 |
| Microbivorous/Detritivorous micro-arthropod abundance^*^ | 0.22 | 0.640 | 0.60 | 0.01 | 0.926 | 0.02 |
| Bacterivorous nematode abundance^*^ | 0.01 | 0.916 | 0.03 | 2.66 | 0.111 | 6.72 |
| Fungivorous nematode abundance^*^ | 0.92 | 0.343 | 2.35 | 1.35 | 0.253 | 3.44 |
| Herbivorous nematode abundance^*^ | 15.5 | **<0.001** | 28.7 | 1.57 | 0.209 | 3.02 |
| Herbivorous epigeal arthropod abundance* | 9.10 | **0.005** | 19.7 | 0.07 | 0.800 | 0.14 |
| *Soil mineral N* |  |  |  |  |  |  |
| NH_4_ concentration^*^ | 13.6 | **0.001** | 11 | 73.3 | **<0.001** | 59.1 |
| NO_3_ concentration^*^ | 5.84 | **0.021** | 8.13 | 29.0 | **<0.001** | 40.4 |
| Total mineral N concentration^*^ | 2.47 | 0.124 | 2.46 | 61.1 | **<0.001** | 60.7 |
| *Plant properties* |  |  |  |  |  |  |
| Graminoid leaf N concentration^*^ | 0.27 | 0.605 | 0.65 | 4.63 | **0.038** | 11.1 |
| Vascular plant shoot biomass^*^ | 0.46 | 0.500 | 1.10 | 4.78 | **0.035** | 11.3 |
| Vascular plant root biomass^*^ | 11.6 | **0.002** | 20.1 | 9.39 | **0.004** | 16.2 |
| Vascular plant root: shoot ratio^*^ | 7.56 | **0.009** | 16.9 | 0.15 | 0.700 | 0.34 |
| Total vascular plant biomass | 7.45 | **0.010** | 12.9 | 13.3 | **0.001** | 23.0 |
| Moss biomass^*^ | 3.20 | 0.082 | 7.38 | 3.22 | 0.081 | 7.42 |
| Graminoid shoot biomass^*^ | 1.10 | 0.301 | 2.30 | 9.68 | **0.004** | 20.3 |
| Forb shoot biomass^*^ | 1.80 | 0.188 | 4.34 | 2.63 | 0.113 | 6.35 |

1. Included organisms which could not be identified into a more specific taxonomic group due to either damage and/or small size [↑](#footnote-ref-1)
